# Nomogram based on dual-energy computed tomography to predict the response to induction chemotherapy in patients with nasopharyngeal carcinoma: a two-center study

**DOI:** 10.1186/s40644-025-00827-7

**Published:** 2025-01-30

**Authors:** Huanhuan Ren, Junhao Huang, Yao Huang, Bangyuan Long, Mei Zhang, Jing Zhang, Huarong Li, Tingting Huang, Daihong Liu, Ying Wang, Jiuquan Zhang

**Affiliations:** 1 Department of Radiology, Chongqing University Cancer Hospital, Chongqing, China; 2 School of Medicine, Chongqing University, Chongqing, China; 3 Department of Radiology, Chongqing General Hospital, Chongqing, China; 4 Radiation Oncology Center, Chongqing University Cancer Hospital, Chongqing, China

**Keywords:** Nasopharyngeal carcinoma, Treatment outcome, Induction chemotherapy, Tomography, X-ray computed, Response

## Abstract

**Background:**

Previous studies utilizing dual-energy CT (DECT) for evaluating treatment efficacy in nasopharyngeal cancinoma (NPC) are limited. This study aimed to investigate whether the parameters from DECT can predict the response to induction chemotherapy in NPC patients in two centers.

**Methods:**

This two-center retrospective study included patients diagnosed with NPC who underwent contrast-enhanced DECT between March 2019 and November 2023. The clinical and DECT-derived parameters of tumor lesions were calculated to predict the response. We employed univariate and multivariate analysis to identify significant factors. Subsequently, the clinical, DECT, and clinical-DECT nomogram models were developed using independent predictors in the training cohort and validated in the test cohort. Receiver operating characteristic analysis was performed to evaluate the models’ performance.

**Results:**

A total of 321 patients were included in the study, predominantly male [247 (76.9%)] with an average age of 52.04 ± 10.87 years. The training cohort (Center 1) comprised 252 patients, while the test cohort (Center 2) comprised 69 patients. Of these, 233 out of 321 patients (72.6%) were responders to induction chemotherapy. The clinical-DECT nomogram showed an AUC of 0.805 (95% CI, 0.688–0.906), outperforming both the DECT model (Extracellular volume fraction [ECVf]) (AUC, 0.706 [95% CI, 0.571–0.825]) and the clinical model (Ki67) (AUC, 0.693 [95% CI, 0.580–0.806]) in the test cohort.

**Conclusions:**

Ki67 and ECVf emerged as independent predictive factors for response to induction chemotherapy in NPC patients. The proposed nomogram, incorporating ECVf, demonstrated accurate prediction of treatment response.

**Supplementary Information:**

The online version contains supplementary material available at 10.1186/s40644-025-00827-7.

## Introduction

Locally advanced nasopharyngeal carcinoma (NPC) is initially diagnosed in approximately 70% of cases [1]. According to NPC treatment guidelines, concurrent chemoradiation is established as the primary treatment modality [2]. Moreover, meta analysis and a Phase III clinical trial have demonstrated that induction chemotherapy can improve survival outcomes compared with concurrent chemoradiotherapy alone in patients with locoregionally advanced NPC [3, 4]. Tumor response to induction chemotherapy independently predicts survival in NPC [5]. However, only 67.6–94.5% NPC patients respond optimally to induction chemotherapy [6]. Therefore, early identification of induction chemotherapy response prior to treatment initiation can facilitate personalized therapeutic approaches, minimizing toxicity and unnecessary costs for non-responders.

Previous studies have endeavored to assess the induction chemotherapy response in NPC patients. Imaging modalities such as positron emission tomography-computed tomography and functional magnetic resonance imaging (MRI) have emerged as promising methods for predicting the response to induction chemotherapy in NPC [7, 8]. However, the widespread application of the two methods is limited. Furthermore, the clinical application of radiomics [9] and deep learning analysis, which require specialized workstations, trained professionals, and extensive preprocessing steps, remains constrained by complexity. Hence, there is an urgent need to identify accurate, user-friendly, and practical markers capable of predicting the induction chemotherapy response in locoregionally advanced NPC.

Previous studies have used DECT to predict neoadjuvant chemotherapy efficacy in lung cancer [10] and hypopharyngeal cancer [11]. Prior studies using DECT parameters to predict induction chemotherapy response in NPC had small sample sizes (under 60) [12, 13], limiting result generalizability and clinical use. Moreover, study results regarding the relationship between iodine concentration and the efficacy of chemotherapy were inconsistent. The extracellular volume fraction (ECVf), derived from DECT, has emerged as a promising predictive factor for non-invasive tumor assessment and has gained recognition in oncological imaging as a valuable marker for predicting post-chemotherapy response [14]. However, limited research has focused on promising DECT parameters for predicting the response to induction chemotherapy in large scale NPC patients.

This study aimed to assess the potential value of DECT in predicting the response to induction chemotherapy and to build a clinical-DECT nomogram integrating DECT parameters and clinical factors to identify optimal candidates who derive maximal benefit from induction chemotherapy in NPC.

## Materials and methods

### Patients

**Fig. 1:**
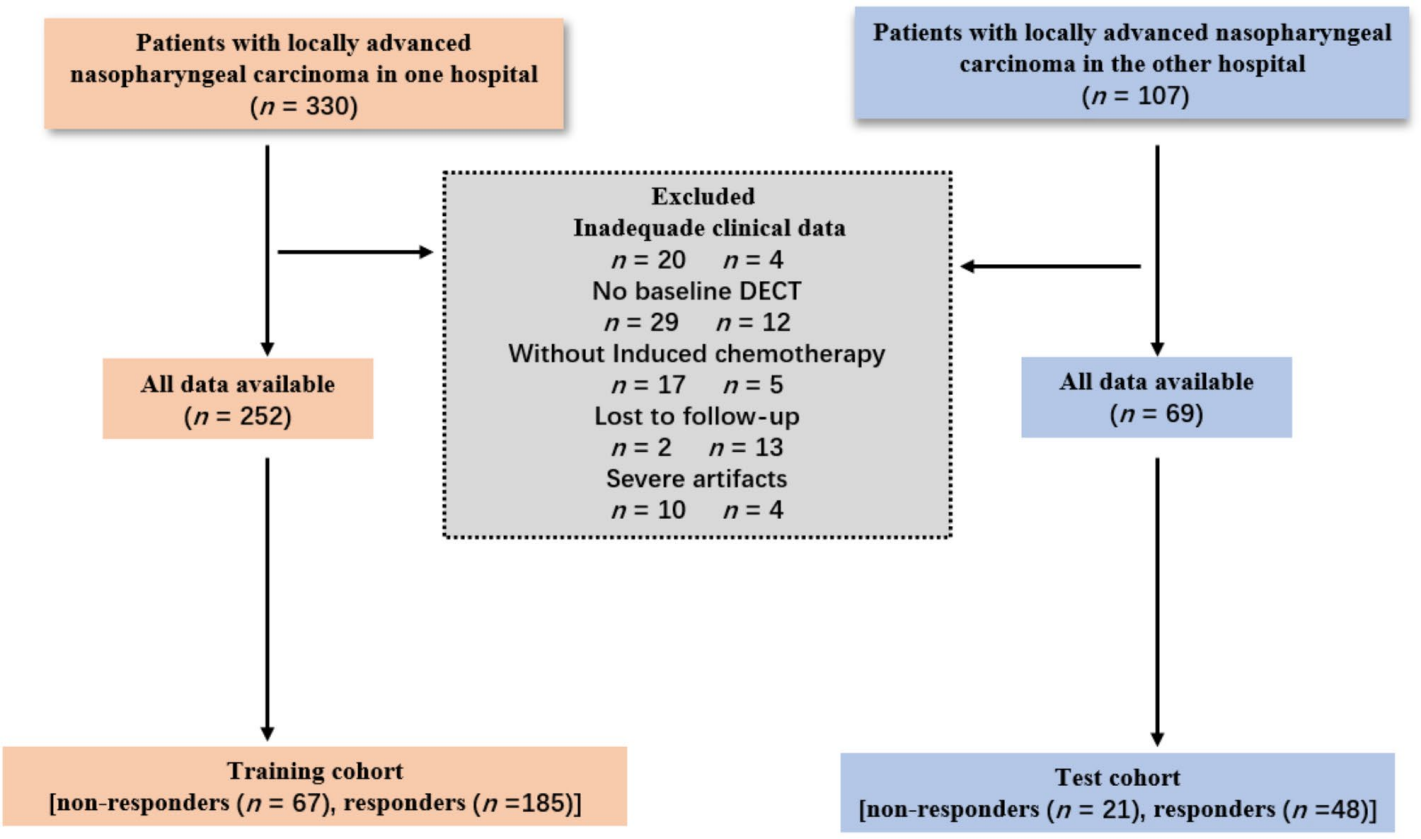
Flowchart of patient selection

This retrospective study received approval from our institutional review board, and the requirement for patients’ informed consent was waived. We consecutively enrolled 321 individuals diagnosed with NPC (252 from center 1 and 69 from center 2) between March 2019 and November 2023. Center 1 patients constituted the training cohort, while those from center 2 formed the test cohort. As shown in Fig. 1, enrollment adhered strictly to the following inclusion criteria: (1) patients had histopathologically confirmed non-keratinizing squamous cell carcinoma, (2) patients completed pretreatment DECT, and (3) patients had stage III or IVA NPC (Eighth American Joint Committee on Cancer [AJCC] staging system) scheduled for induction chemotherapy. Patients who (1) received anti-tumor treatment before DECT, (2) lacked induction chemotherapy, (3) had inadequate image quality/data, or (4) were lost to follow-up were excluded.

Demographic characteristics and laboratory data were independently reviewed from the electronic medical records system of each center.

### Acquisition and postprocessing of DECT images

The scan parameters of the two centers are detailed in the Supplementary Materials.

Two experienced radiologists from center 1 (with 7 and 12 years in head and neck radiology) independently reviewed all images while blinded to clinical and pathological data. The regions of interest (ROIs) from the largest axial slices of nasopharyngeal primary tumors encompassing the tumor contour were manually delineated. The ROIs were carefully delineated to include primary lesions and exclude areas of necrosis, cyst, and hemorrhage [12]. Then, the ROIs were automatically transferred to virtual monoenergetic images at 40 keV and 80 keV, iodine concentration maps, effective atomic number (Zeff) maps, and electron density (ED) maps. The values of these quantitative parameters were obtained in both non-contrast and venous phases.

The following quantitative parameters were calculated in the venous phase: (1) the slope of the spectral curve, where slope = (HU_40 keV_ − HU_80 keV_)/40; (2) the normalized iodine concentration (NIC) value, where NIC = IC_tumor_/IC_internal carotid_ [13] (IC_tumor,_ is the iodine concentration of the tumor; IC_internal carotid_ is the iodine concentration of the internal carotid at the level of the tumor); and (3) the ECVf of the tumor, where ECVf = (1 − hematocrit) × NIC × 100% [14]. The interval time between the DECT scan and hematocrit measurement was 1.0 ± 1.3 days. The radiologists mentioned previously performed measurements for all the parameters. The mean values of each parameter measured by the two radiologists were used for statistical analysis.

### Treatment procedure and evaluation of the tumor response to induction chemotherapy

The interval time between the DECT scan and induction chemotherapy was 3.1 ± 1.9 days. The response to induction chemotherapy was assessed after two or three cycles of chemotherapy in both institutions. Induction chemotherapy regimens included GP (gemcitabine 1000 mg/m^2^, days 1 and 8; cisplatin 80 mg/m^2^, day 1), TPC (paclitaxel 135–175 mg/m^2^, day 1; cisplatin 80 mg/m^2^, day 1; capecitabine 1000 mg/m^2^ twice per day, days 1–14), TP (paclitaxel 135–175 mg/m^2^, day 1; cisplatin 80 mg/m^2^, day 1), PF (cisplatin 80 mg/m^2^, day 1; fluorouracil 750 mg/m^2^, days 1–5), TPF (paclitaxel 135–175 mg/m^2^, day 1; cisplatin 80 mg/m^2^, day 1; fluorouracil 750 mg/m^2^, days 1 − 5), DPF (docetaxel 75 mg/m^2^ and cisplatin 80 mg/m^2^, day 1; fluorouracil 750 mg/m^2^, days 1–5), DP (docetaxel 75 mg/m^2^ and cisplatin 80 mg/m^2^, day 1), and DPC (docetaxel 75 mg/m^2^ and cisplatin 80 mg/m^2^, day 1; capecitabine 1000 mg/m^2^ twice per day, days 1–14).

Based on the Response Evaluation Criteria in Solid Tumors (RECIST) version 1.1 [15], the treatment response of all patients was evaluated using MRI (on T2-weighted imaging with fat saturation images and contrast-enhanced T1-weighted imaging images). Following the RECIST criteria, complete responses were delineated as tumor disappearance, while partial responses were characterized by a reduction in tumor diameter of ≥ 30%. An augmentation of ≥ 20% in tumor diameter was designated as progressive disease. All other scenarios were classified as stable disease. Responders were designated as patients achieving complete or partial responses, while non-responders were considered those experiencing stable or progressive disease.

### Development of predictive models

Univariate logistic regression analysis was used to select important clinical variables and DECT parameters to predict the response to induction chemotherapy in NPC patients. Thereafter, clinically significant features (*P* < 0.05) were selected to identify independent predictors of the response to induction chemotherapy by stepwise backward multivariate analysis. Independent clinical factors and DECT predictors were then used to construct the clinical model and the DECT model, respectively, through multivariate logistic regression analysis. Ultimately, the clinical-DECT nomogram was built, integrating the significant clinical and DECT predictive factors. The three models were then independently verified in the test cohort.

### Evaluation of predictive models

Receiver operating characteristic (ROC) curves were generated for each model to assess predictive performance, with the area under the ROC curve (AUC) calculated accordingly. The DeLong method was used to compare AUC values. Calibration curves were generated to assess the calibration performance of the three models, facilitating the comparison of the agreement between the observed clinical outcomes and the predictive outcomes. Subsequently, decision curve analysis was performed to evaluate the clinical usefulness of these predictive models.

### Statistical analysis

Statistical analysis was performed with R (version 4.3.1; R Foundation for Statistical Computing) and SPSS software (version 27.0; IBM Corporation). Continuous variables are expressed as mean and standard deviation values, and categorical variables are expressed as numbers and percentages. Student’s *t* test or the Wilcoxon rank-sum test was used for patients’ age, body mass index, neutrophil-to-lymphocyte ratio, platelet-to-lymphocyte ratio, systemic immune inflammation index, and platelet count. Patient sex, smoking and drinking history, T stage, N stage, AJCC stage, Epstein–Barr virus (EBV) DNA, and Ki67 status were compared using χ^2^ or Fisher exact tests. Both the intra- and interobserver reproducibility of the DECT parameters (Zeff_NCCT_, Zeff_V_, ED_NCCT_, ED_V_, Slope, NIC, and ECVf) were evaluated using the intraclass correlation coefficient. ROC curve analysis was used to evaluate the predictive performances of the clinical, DECT, and clinical-DECT nomogram models. Two-sided *P* values of < 0.05 were considered statistically significant.

## Results

### Patient characteristics

The characteristics of the 321 NPC patients [male: 247 (76.9%); average age: 52.04 ± 10.87] are shown in Table 1. Some clinical features were not included in this study due to a high proportion of missing data (Supplementary Material). As shown in Fig. 1, there were 185 responders and 67 non-responders in the training cohort and 48 responders and 21 non-responders in the test cohort.

**Table 1:** Characteristics of participants

Characteristics	Overall			Training cohort (*n* = 252)		*p*	Test cohort (*n* = 69)		*p*
	(*n* = 321)		Non-response (*n* = 67)		Response (*n* = 185)		Non-response (*n* = 21)	Response (*n* = 48)	
Age (mean ± SD, years)	52.04 ± 10.87		52.80 ± 12.86		51.80 ± 10.54	0.536	48.95 ± 10.65	53.29 ± 9.20	0.09
Sex (%)						0.615			1.000
Male	247 (76.9)		48 (71.6)		138 (75.0)		19 (90.5)	42 (87.5)	
Female	74 (23.1)		19 (28.4)		47 (25.0)		2 (9.5)	6 (12.5)	
Smoking (%)						0.48			0.003
No	235 (73.2)		52 (77.6)		132 (71.4)		21 (100.0)	30 (62.5)	
Yes	86 (26.8)	15 (22.4)			53 (28.6)		0 (0.0)	18 (37.5)	
Drinking (%)						0.023			0.545
No	259 (80.7)	59 (88.1)			142 (76.8)		19 (90.5)	39 (81.2)	
Yes	62 (19.3)	8 (11.9)			43 (23.2)		2 (9.5)	9 (18.8)	
T stage (%)						0.207			0.014
1	20 (6.23)	4 (5.97)			9 (4.86)		3 (14.29)	4 (8.33)	
2	82 (25.55)	13 (19.4)			54 (29.19)		3 (14.29)	12 (25.00)	
3	132 (41.12)	33 (49.25)			68 (36.76)		11 (52.38)	13 (27.08)	
4	87 (27.10)	17 (25.37)			54 (29.19)		4 (19.05)	19 (39.58)	
N stage (%)						0.849			0.483
0	20 (6.2)	5 (7.5)			9 (4.9)		2 (9.5)	4 (8.3)	
1	127 (39.6)	24 (35.8)			68 (36.8)		12 (57.1)	23 (47.9)	
2	119 (37.1)	26 (38.8)			70 (37.8)		7 (33.3)	16 (33.3)	
3	55 (17.1)	12 (17.9)			38 (20.5)		0 (0.0)	5 (10.4)	
AJCC (%)						0.668			0.239
III	152 (47.4)	35 (52.2)			85 (45.9)		12 (57.1)	20 (41.7)	
IVA	169 (52.6)	32 (47.8)			100 (54.1)		9 (42.9)	28 (58.3)	
EBV DNA (copies/mL)						0.09			0.557
0-999	123 (38.3)	30 (44.8)			66 (35.7)		8 (38.1)	19 (39.6)	
1000–10,000	90 (28.0)	13 (19.4)			60 (32.4)		4 (19.0)	13 (27.1)	
10,000–100,000	53 (16.5)	8 (11.9)			33 (17.8)		3 (14.3)	9 (18.8)	
> 100,000	55 (17.1)	16 (23.9)			26 (14.1)		6 (28.6)	7 (14.6)	
Ki67 (%)						< 0.001			0.002
< 50%	152 (47.4)	48 (71.6)			69 (37.3)		17 (81.0)	18 (37.5)	
≥ 50%	169 (52.6)	19 (28.4)			116 (62.7)		4 (19.0)	30 (62.5)	
BMI (mean ± SD)	23.06 ± 4.32	23.58 ± 3.02			22.78 ± 4.89	0.22	23.53 ± 3.70	23.24 ± 3.71	0.76
NLR (mean ± SD)	2.93 ± 1.54	2.99 ± 1.51			2.85 ± 1.52	0.526	3.56 ± 2.32	2.85 ± 1.24	0.102
PLR (mean ± SD)	161.72 ± 79.41	167.31 ± 98.86			155.50 ± 69.20	0.294	190.17 ± 103.73	165.65 ± 73.91	0.268
Note: Data are shown as mean and standard deviation values, median and interquartile range values, or numbers and percentages in parenthesis. BMI = body mass index; CI = confidence interval; EBV DNA = Epstein–Barr virus DNA; NLR = neutrophil-to-lymphocyte ratio; PLR = platelet-to-lymphocyte ratio.									

The inter- and intra-observer agreements of DECT parameters within primary tumors ranged from 0.754 to 0.904 (Supplementary Material).

### Predictors of induction chemotherapy response in NPC patients

Following univariate and stepwise backward multivariate analysis, Ki67 (odds ratio [OR], 2.880; 95% confidence interval [CI], 1.687–4.915; *P* < 0.001] and ECVf (OR, 0.706; 95% CI, 0.542–0.918; *P* = 0.009) emerged as independent predictors of the treatment response to induction chemotherapy (Table 2).

**Table 2:** Univariate and stepwise backward multivariate analysis of characteristics associated with induction chemotherapy response in the training cohort

Characteristics		Univariable	*p*			Multivariable	*p*
		Odds Ratio (95% CI)				Odds Ratio (95% CI)	
Age		0.911 (0.686, 1.212)	0.523				
Sex							
Male	Reference						
Female	0.770 (0.415, 1.430)		0.408				
Smoking							
No	Reference						
Yes	1.304 (0.676, 2.517)		0.428				
Drinking							
No	Reference						
Yes	2.162 (1.027, 4.552)		0.042*				
BMI	0.833 (0.610, 1.138)		0.252				
NLR	0.918 (0.697, 1.209)		0.544				
PLR	0.858 (0.656, 1.123)		0.265				
SII	1.037 (0.779, 1.381)		0.803				
Platelet (10^9^ /L)	0.995 (0.751, 1.319)		0.974				
Ki-67 status							
<50%	Reference						
≥50%	2.808 (1.659, 4.754)		**<** 0.001*		2.880 (1.687, 4.915)		**<** 0.001*
T stage							
T1	Reference						
T2	0.751 (0.419, 1.346)		0.336				
T3	0.618 (0.345, 1.106)		0.105				
T4	1.124 (0.592, 2.134)		0.722				
N stage							
N0	Reference						
N1	0.960 (0.528, 1.746)		0.894				
N2	1.079 (0.592, 1.968)		0.804				
N3	1.175 (0.558, 2.472)		0.671				
AJCC							
III	Reference						
IVa	1.092 (0.612, 1.948)		0.767				
EBV DNA (copies/mL)							
0-999	0.669 (0.379, 1.183)		0.167				
1000–10,000	1.863 (0.944, 3.675)		0.073				
10,000–100,000	1.479 (0.645, 3.390)		0.355				
>100,000	Reference						
Zeff_NCCT_	1.109 (0.837, 1.470)		0.471				
Zeff_V_	0.803 (0.609, 1.061)		0.123				
ED_NCCT_	0.781 (0.587, 1.038)		0.088				
ED_V_	0.807 (0.608, 1.074)		0.141				
Slope	0.766 (0.580, 1.010)		0.059				
NIC	0.793 (0.615, 1.021)		0.029*				
ECVf	0.718(0.555, 0.928)		0.011*		0.706 (0.542, 0.918)		0.009*

Note: Data in parentheses are 95% CI values. BMI = body mass index; CI = confidence interval; EBV DNA = Epstein–Barr virus DNA; ECVf = (1 − hematocrit) × NIC × 100%; ED_NCCT_ = electron density in non-contrast DECT images; ED_V_ = electron density in venous phase; NIC = normalized iodine concentration in venous phase; NLR = neutrophil-to-lymphocyte ratio; PLR = platelet-to-lymphocyte ratio; SII = systemic immune inflammation index; Slope = (HU_40 keV_ − HU_80 keV_)/40; Zeff_NCCT_ = effective atomic number in non-contrast DECT images; and Zeff_V_ = effective atomic number in venous phase

* Indicates statistical significance; *P* < 0.05

### Predictive performances of the clinical and DECT models

**Fig. 2:**
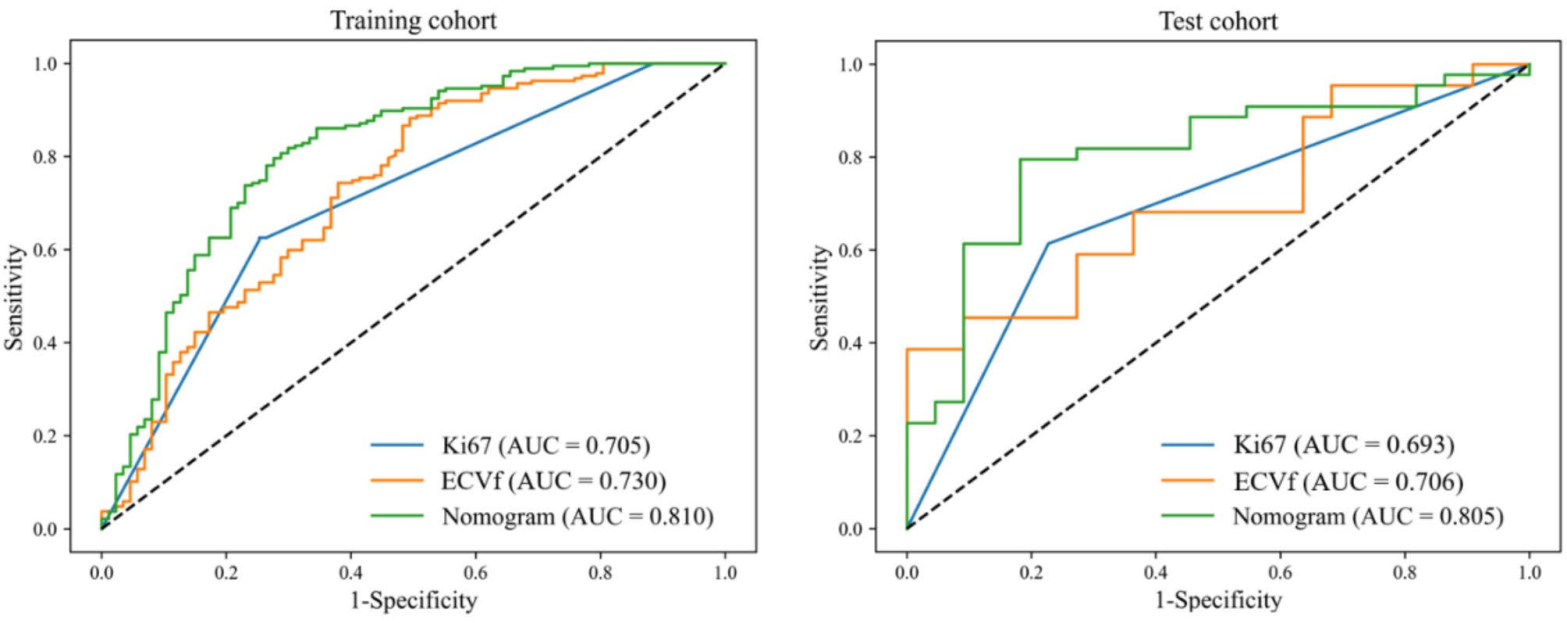
ROC curves of the Ki67, ECVf, and nomogram models for predicting the response to induction chemotherapy in the training and test cohorts. Note: ECVf = extracellular volume fraction; ROC = receiver operating characteristic

In delineating patients classified as responders to induction chemotherapy, the clinical model (Ki67) had an AUC of 0.705 (95% CI, 0.646–0.759) in the training cohort and an AUC of 0.693 (95% CI, 0.580–0.806) in the test cohort (Table 3; Fig. 2).

In the training cohort, the DECT model (ECVf) exhibited an AUC of 0.730 (95% CI, 0.665–0.794), while in the test cohort, it showed an AUC of 0.706 (95% CI, 0.571–0.825) (Table 3; Fig. 2). The DeLong test revealed no significant difference in AUCs between the clinical model (Ki67) and the DECT model (ECVf) in both the training cohort (*P* = 0.591, DeLong test) and the test cohort (*P* = 0.881, DeLong test) (Table 4).

**Fig. 3:**
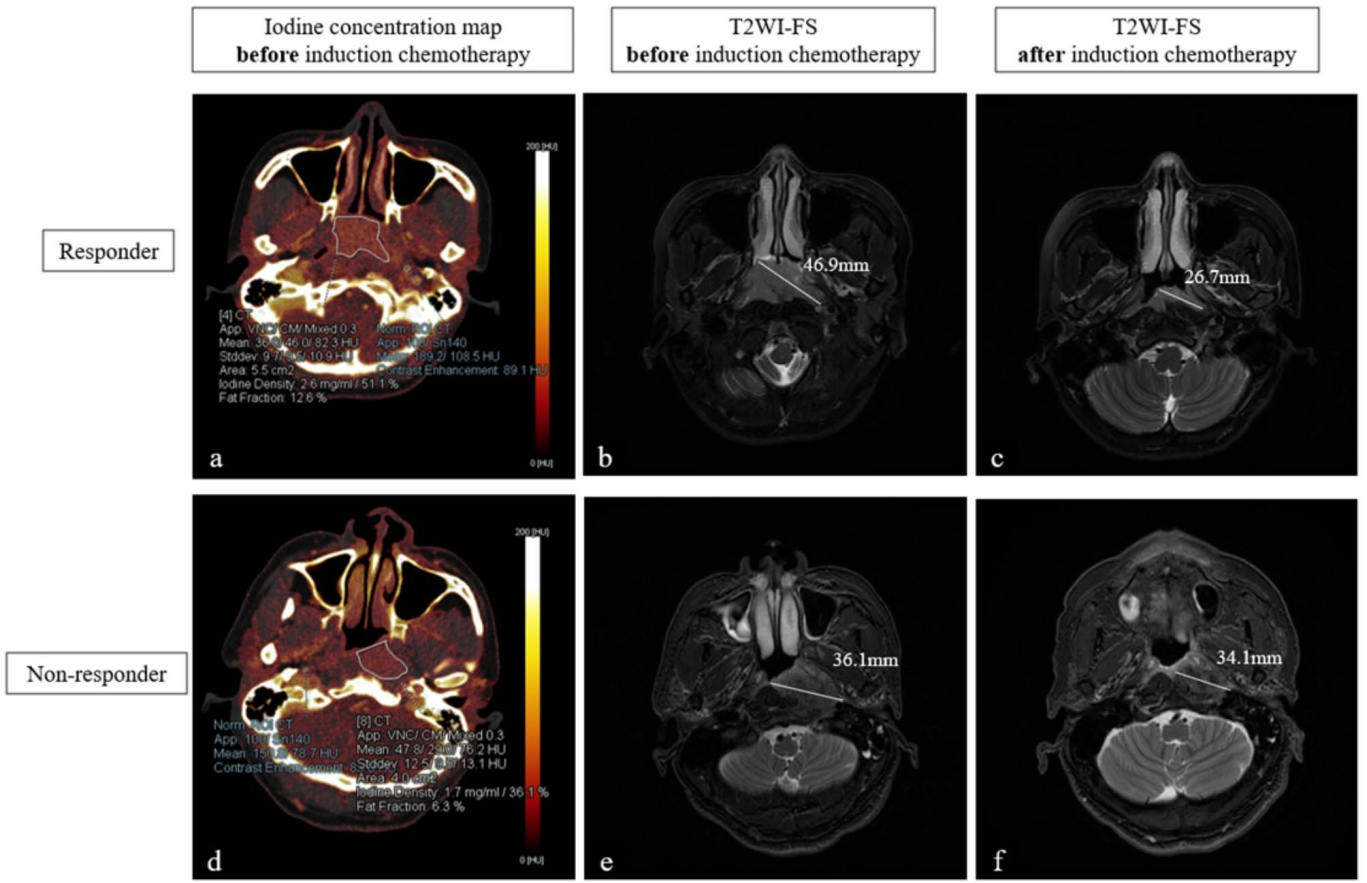
Representative images of two patients with NPC after induction chemotherapy. Note: The upper row of images shows the iodine map (**a**) and T2WI-FS (**b**) of the nasopharynx of a 51-year-old male patient with NPC (Ki67 60% [+], T3N1M0) before induction chemotherapy as well as T2WI-FS (**c**) after two cycles of induction chemotherapy (paclitaxel 180 mg, day 1; nedaplatin 80 mg, day 1; capecitabine 1.5 g twice per day, days 1–14). According to RECIST 1.1, this patient was recognized as achieving a partial response and classified into the responder group. Two radiologists measured the normalized iodine concentration of the maximum slice and adjacent upper and lower slices of the ROI and then calculated the average ECVf to be 28.6% The lower row of images shows the iodine map (**d**) and T2WI-FS (**e**) of the nasopharynx of a 58-year-old male patient with NPC (Ki67 10% [+], T3N2M0) before treatment as well as T2WI-FS (**f**) after two cycles of induction chemotherapy (paclitaxel 240 mg, day 1; nedaplatin 105 mg, day 1). According to RECIST 1.1, this patient was recognized as having stable disease and classified into the non-responder group. The average ECVf obtained using the same method mentioned above was 37.0%

Two representative DECT images are displayed in Fig. 3, one of which is from a responder and the other of which is from a non-responder.

**Table 3:** Performances of the Ki67, ECVf, and nomogram models for predicting induction chemotherapy response in the training and test cohorts

Cohorts	Models	AUC	Sen	Spe	PPV	NPV
Training cohort	Ki67	0.705 (0.646, 0.759)	0.626	0.747	0.842	0.481
	ECVf	0.730 (0.665, 0.794)	0.882	0.506	0.793	0.667
	Nomogram	0.810 (0.751, 0.866)	0.797	0.724	0.861	0.624
Test cohort	Ki67	0.693 (0.580, 0.806)	0.614	0.773	0.844	0.500
	ECVf	0.706 (0.571, 0.825)	0.604	1.000	1.000	0.525
	Nomogram	0.805 (0.688, 0.906)	0.795	0.818	0.897	0.667

Note: ECVf = extracellular volume fraction; Nomogram, the model combining Ki67 and ECVf from dual-energy CT; NPV = negative predictive value; PPV = positive predictive value; Sen = sensitivity; and Spe = specificity

**Table 4:** Comparison of the performances of the Ki67, ECVf, and nomogram models for predicting induction chemotherapy response in the training and test cohorts

Cohorts	Models	Ki67	ECVf	Nomogram
Training cohort	Ki67	NA	0.591	< 0.001
	ECVf	0.591	NA	0.023
	Nomogram	< 0.001	0.023	NA
Test cohort	Ki67	NA	0.880	0.006
	ECVf	0.880	NA	0.139
	Nomogram	0.006	0.139	NA

Note: The numbers in the table are the *p*-values of Delong test. Note: ECVf = extracellular volume fraction; Nomogram, the model combining Ki67 and ECVf from dual-energy CT

### Predictive performance of the clinical-DECT nomogram model

**Fig. 4:**
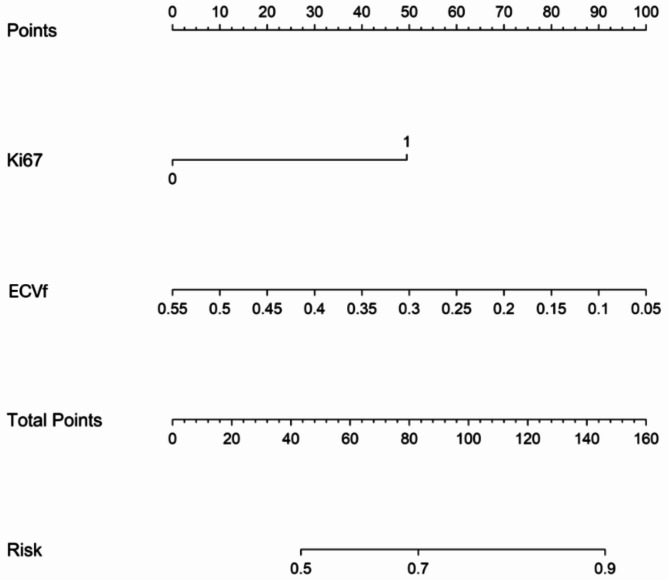
Nomogram for predicting the response to induction chemotherapy among NPC patients. Note: ECVf = extracellular volume fraction

**Fig. 5:**
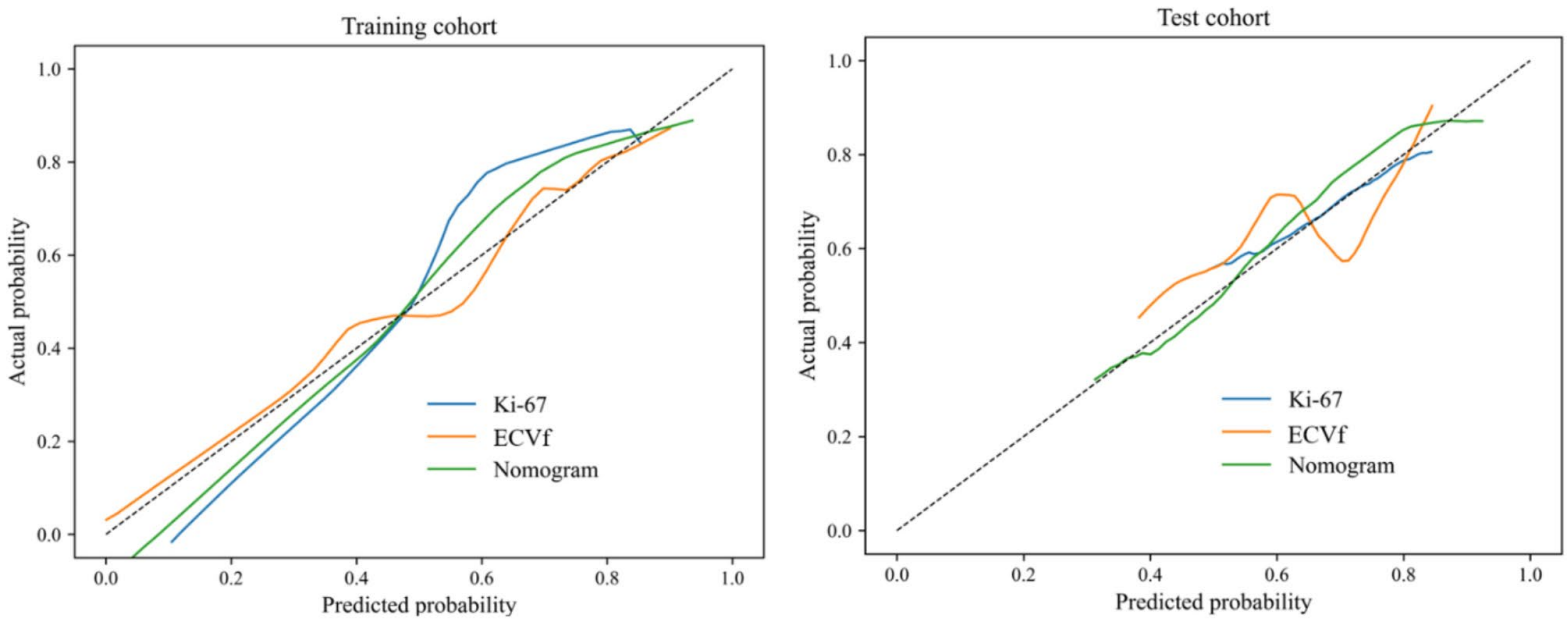
Calibration curves for the Ki67, ECVf, and models in the training and test cohorts. Note: ECVf = extracellular volume fraction

The combined model, termed the clinical-DECT nomogram, which was designed to identify responders to induction chemotherapy in the training cohort, is presented in Fig. 4. In this cohort, the clinical-DECT nomogram showcased an AUC of 0.810 (95% CI, 0.751–0.866), while in the test cohort, it yielded an AUC of 0.805 (95% CI, 0.688–0.906) (Table 3; Fig. 2). The DeLong test indicated the clinical-DECT nomogram have significantly enhanced predictive performance compared with the clinical model (Ki67) in both the training cohort (null hypothesis: AUC of the clinical-DECT nomogram = AUC of the clinical model; *P* < 0.001) and the test cohort (null hypothesis: AUC of the clinical-DECT nomogram = AUC of the clinical model; *P* = 0.006). The DeLong test indicated that the clinical-DECT nomogram outperformed the DECT model (ECVf) in the training cohort (null hypothesis: AUC of the clinical-DECT nomogram = AUC of the DECT model; *P* = 0.023), whereas no significant difference was observed in the test cohort (null hypothesis: AUC of the clinical-DECT nomogram = AUC of the DECT model; *P* = 0.139) (Table 4). The calibration curve indicated favorable concordance between the predicted probability of the induction chemotherapy response by the clinical-DECT nomogram and the observed response in both the training cohort (null hypothesis: the clinical-DECT nomogram has good fit; *P* = 0.074, Hosmer–Lemeshow test) and the test cohort (null hypothesis: the clinical-DECT nomogram has good fit; *P* = 0.058, Hosmer–Lemeshow test) (Fig. 5).

**Fig. 6:**
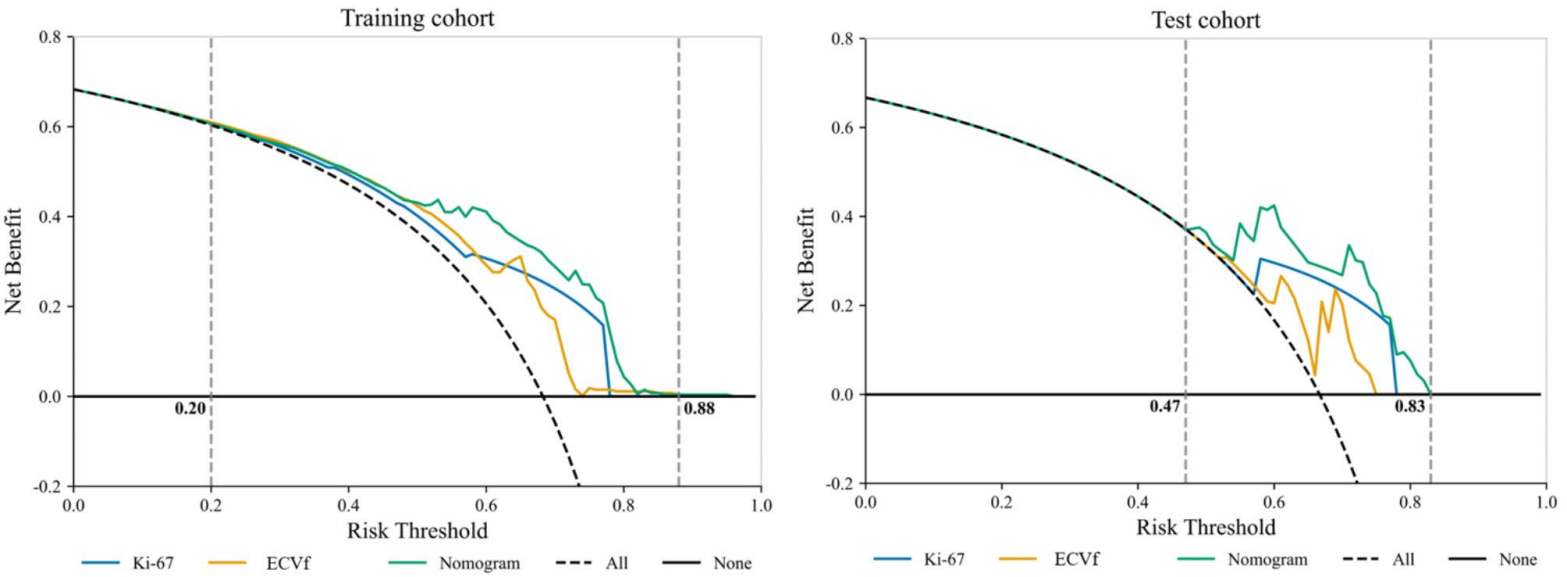
Decision curve analysis of the Ki67, ECVf, and nomogram models in the training and test cohorts. Note: ECVf = extracellular volume fraction

The decision analysis curves indicated that the three models showed superior capability to predict the response to induction chemotherapy in NPC patients compared with the treat-all-patients and treat-none approaches in both the training and test cohorts. Generally, the clinical-DECT nomogram exhibited superior performance in comparison to the Ki67 and ECVf models, with clinical net benefits consistently being > 0, ranging from 0.47 to 0.83 in the test cohort (Fig. 6).

## Discussion

In this study, the clinical, DECT, and clinical-DECT models were developed to predict the response to induction chemotherapy in NPC patients. Our results suggest that the clinical-DECT nomogram can more accurately select patients suitable for induction chemotherapy than either the clinical or the DECT model.

ECV represents the sum of extravascular–extracellular and intravascular spaces, with tumor aggressiveness and treatment response being influenced by the ECV [16, 17]. Some investigations into pancreatic cancer [18] and breast cancer [19] have revealed an inverse relationship between ECVf levels and chemotherapy efficacy. Our study indicated that the pretreatment ECVf of NPC is an independent predictor of post-chemotherapy response. Potential explanations are as follows: (1) increased levels of fibroblasts, particularly cancer-associated fibroblasts, within the tumor microenvironment, which may promote chemotherapy resistance, as observed in pancreatic [18], colorectal [20], and lung cancers [21]; (2) extracellular matrix remodeling leading to tumor hypoxia, thereby impeding drug penetration and reducing therapeutic efficacy, as demonstrated in colon [22] and pancreatic cancers [23]; and (3) a robust correlation between ECVf and desmoplastic stroma, as demonstrated by previous research [24]. The desmoplastic stroma has been implicated in facilitating epithelial–mesenchymal transition and diminishing drug perfusion, thus potentially contributing to chemotherapy resistance [25, 26]. In the study, RECIST 1.1 was used as the reference standard for assessing chemotherapy response; however, the criteria may not fully capture the impact of the diverse biological characteristics of NPC. Our findings suggest that ECVf, as a functional parameter derived from DECT, can provide information on the extracellular matrix, reflecting the state of the tumor microenvironment. Thus, ECVf has the potential to serve as a supplementary indicator for assessing treatment response.

Previous studies have highlighted the significance of NIC in predicting induction chemotherapy efficacy in NPC patients [12, 13]. Our outcome differed from those of Zhan [12] and Liao [13]. The patient population included in these three studies was heterogeneous due to the incidence rate of NPC in various geographic areas, individual characteristics of patients, varying experience levels of physicians, and the use of a wide array of chemotherapy regimens. All of these factors may have led to differences in the outcomes of these studies. In our study, the multivariate analysis did not identify NIC as a significant factor. Liao’s study found pretreatment higher NIC levels in responders than in non-responders among NPC patients [13]. They hypothesized that elevated NIC could enhance treatment efficacy by improving the local oxygen supply and drug concentration. Conversely, Zhan’s results contradict this, suggesting that greater iodine concentrations in lesions might impede local blood flow and perfusion, thereby hampering drug delivery and therapeutic efficacy [12]. Although these results are inconsistent, our study stands out due to its large sample size, significant patient heterogeneity, and diverse chemotherapy regimens, closely reflecting real-world medical scenarios. Furthermore, the robustness and generalizability of our findings were ensured through external validation.

Furthermore, our study supports the notion that tumors with higher levels of Ki67 status exhibit better responses to chemotherapy than those with lower Ki67 status. Ki67 serves as an important marker of tumor cell proliferation activity. Chemotherapy agents could effectively inhibit highly proliferative cells and impede tumor progression. Previous studies have highlighted the correlation between high Ki67 levels and enhanced responsiveness to neoadjuvant chemotherapy in breast cancer [27, 28]. These findings indicate that highly proliferating tumors are more susceptible to chemotherapy.

Previous studies have identified that changes in EBV DNA copy number before or during treatment can predict the therapeutic response and prognosis in NPC patients [29]. However, our results indicate that the pre-treatment EBV DNA copy number is not an independent predictive factor for the efficacy of induction chemotherapy, which is consistent with the findings of several other studies [30]. The discrepancies in these results may be attributed to differences in the equipment, methods, or cutoff values used for measuring EBV DNA copy numbers. Therefore, standardization of the measurement of this parameter is necessary in future research.

To facilitate clinical utility, the clinical-DECT nomogram combining ECVf and Ki67 was developed to predict the response to induction chemotherapy in NPC patients. This nomogram was well calibrated and exhibited a superior discriminative ability relative to that of either Ki67 or ECVf alone. Recent studies have demonstrated the ability of CT-based deep learning (AUC, 0.811) or MRI-based radiomics (C-index, 0.863) to predict induction chemotherapy response in patients with locally advanced NPC [31, 32]. However, the implementation of radiomics models in clinical practice is hindered by the complexity of feature extraction and the requirements for preprocessing across multiple CT phases or MRI sequences. Our nomogram demonstrated diagnostic efficacy comparable to that of previous models, with the added benefits of reduced scanning times and simplified parameter calculation procedures. Additionally, we developed an interactive online platform (https://huangyao96.shinyapps.io/DynNomapp/) to estimate the probability of the induction chemotherapy response in new patients. If the probability is high, the treatment can proceed as initially planned. Conversely, if the probability is low, clinicians can escalate the induction chemotherapy regimen prior to initiation or proceed directly with CCRT, thus avoiding unnecessary expensive chemotherapy regimens and their toxic effects, expense, and a prolonged waiting period before CCRT [33].

This study has several limitations. First, our data, including the training and test cohorts, came from Siemens equipment, so whether the results apply to data from other manufacturers still needs further validation. Second, ECVf measurement using the venous phase at 60–70 s after contrast administration might not allow sufficient time for contrast agent equilibration. However, extending measurement to the late equilibrium phase can disrupt routine clinical workflow, and the minimum scan delay time for reliable ECVf estimation remains undefined. Third, the inclusion of patients with varying stages and chemotherapy regimens warrants future subgroup analyses to assess chemotherapy efficacy across different demographics. Nevertheless, our findings have practical implications for clinical settings. Lastly, owing to the significant imbalance between the effective and ineffective groups (approximately 99:1) following chemoradiotherapy for NPC patients at our institution, we did not consider the efficacy of chemoradiotherapy as a clinical endpoint. Due to the limited duration of our follow-up, prognosis evaluation was not feasible in this study. Future studies should perform in-depth baseline and follow-up data collection to determine the utility of DECT parameters in chemoradiotherapy response and prognostic evaluation of NPC.

In conclusion, pretreatment ECVf derived from DECT can serve as an objective imaging marker for predicting the induction chemotherapy response in patients with NPC. The distinguishing performance of a nomogram combining ECVf and Ki67 for predicting the therapeutic effect is especially valuable to clinicians to help facilitate personalized treatment decisions.

## Electronic supplementary material

Below is the link to the electronic supplementary material.

Supplementary Material 1

## Data Availability

The datasets generated and/or analyzed during the current study are not publicly available because the subjects did not provide written consent for their data to be publicly shared. However, datasets can be obtained by request from specific research groups.

## Abbreviations

AUC: Area under the receiver operating characteristic curve
CI: Confidence interval
DECT: Dual-energy computed tomography
ECVf: Extracellular volume fraction
ED: Electron density
MRI: Magnetic resonance imaging
NIC: Normalized iodine concentration
NPC: Nasopharyngeal carcinoma
ROI: Regions of interest
Zeff: Effective atomic number
