# Supplementary material for: Nomogram based on dual-energy computed tomography to predict the response to induction chemotherapy in patients with nasopharyngeal carcinoma: a two-center study
Source: Cancer Imaging. 2025 Jan 30;25:8. doi: 10.1186/s40644-025-00827-7 (PMC11781003; doi:10.1186/s40644-025-00827-7)
Supplement: Supplementary file 1 — Supplementary Material 1 [file 40644_2025_827_MOESM1_ESM.docx]

**Supplemental Materials**

**Materials and methods**

**Dual-energy computed tomography (DECT) image acquisition and postprocessing**

In center 1, dual-phase (non-contrast CT and venous phase) scans of nasopharynx of 252 participants were acquired with a DECT scanner (SOMATOM Drive, Siemens Healthineers). The tube voltages were set at 100 kVp and Sn140 kVp (adding the tin filter). Advanced model-based iterative reconstruction (strength, 3) and automatic current modulation (CARE Dose 4D, Siemens Healthineers) were used. Iodine contrast media (Loversol, 320 mg/mL, HENGRUI Medicine) was administered through the right or left ulnar vein by a dual-head injector. The dosage was 1.5 mL/kg, and the flow rate was 2.5 mL/s, followed by 20 mL of 0.9% normal saline administered at the same flow rate. The venous phase began 60 s after contrast injection.

In center 2, dual-phase (non-contrast CT and venous phase) scans of nasopharynx of 69 participants were acquired with a DECT scanner (SOMATOM Force, Siemens Healthineers). The scanning parameters were as follows: the tube voltages were set at 100 kVp and Sn140 kVp. CARE Dose 4D (Siemens Healthineers) was used; detector collimation, 128 × 0.6 mm; reconstruction thickness, 1.0 mm. Contrast material (Iopromide, 370 mg/mL, Bayer) was injected at a rate of 3.5 mL/s (1.0 mL/kg of body weight). The venous phase began 60 − 70 s after contrast injection.

The postprocessing of DECT images from the two institutions was performed on a syngo.via workstation (syngo.via VB40A, Dual Energy, Siemens Healthineers). Transverse sections were reconstructed for all series using the following parameters: thickness, 1.5 mm; increment, 1.2 mm; and soft-tissue kernel, J30f. These series were then transmitted to the local picture archiving and communication systems.

**Results**

**Patient characteristics**

The following characteristic parameters were excluded due to significant proportions of missing data: dehydrogenase, epidermal growth factor receptor, Eastern Cooperative Oncology Group performance status, and patient-generated subjective global assessment, with missing proportions of 60.75% (195/321), 67.60% (217/321), 40.19% (129/321), and 79.75% (256/321), respectively.

**Inter- and intra-observer agreement**

Inter-observer and intra-observer reproducibility analysis was performed on 30 patients randomly selected from center 1. The regions of interest (ROIs) of each patient were delineated again by the same radiologist after 30 days interval and by another radiologist using the same method. As to the intra- and interobserver reproducibility of ROI delineation, the intra-observer ICC for Zeff_NCCT_, Zeff_V_, ED_NCCT_, ED_V_, slope, normalized iodine concentration (NIC), and extracellular volume fraction (ECVf) for the first radiologist were 0.783, 0.849, 0.754, 0.826, 0.873, 0.878, and 0.904. Meanwhile, the inter-observer agreement for Zeff_NCCT_, Zeff_V_, ED_NCCT_, ED_V_, slope, NIC, and ECVf between the two radiologists were 0.776, 0.843, 0.785, 0.809, 0.847, 0.836, and 0.892, respectively. The results indicated good or excellent intra- and interobserver ROI delineation reproducibility.
